# Rapid and reagent-free screening of occult hepatitis B virus infection based on plasma Vis-NIR spectral pattern recognition

**DOI:** 10.3389/fbioe.2026.1774782

**Published:** 2026-02-23

**Authors:** Linbin Huang, Xiaoting Huang, Jingjing Xia, Lining Huang, Huanjie Zhou, Min Chen, Baoren He, Meijun Chen, Qiuhong Mo, Tao Pan, Chao Ou

**Affiliations:** 1 Guangxi Medical University Cancer Hospital, Nanning, China; 2 Department of Optoelectronic Engineering, Jinan University, Guangzhou, China; 3 Nanning Blood Center, Nanning, China

**Keywords:** blood screening, multi-wavelength, norris derivative filtering, occult hepatitis B virus infection, partial least squares-discriminant analysis, separation degree priority combination, step-by-step phase-out, visible-near-infrared spectralpattern recognition

## Abstract

**Introduction:**

Occult hepatitis B virus infection (OBI) is a specific form of hepatitis B virus (HBV) infection characterized by testing negative for Hepatitis B surface antigen (HBsAg) with the presence of HBV DNA in the blood. Due to the complexity and high cost of HBV DNA testing, which is rarely included in routine physical examinations, leading to underdiagnosis of OBI. In this study, plasma visible-near-infrared (Vis-NIR) spectroscopy pattern recognition was employed to develop the discriminant analysis models for distinguishing between OBI from healthy (normal controls) plasma.

**Methods:**

A total of 444 plasma samples from voluntary blood donors (OBI 204, normal controls 240) were collected, and their Vis-NIR spectra were measured. The samples were rigorously divided into training, prediction, and independent external validation sets. Partial least squares-discriminant analysis (PLS-DA) and k-nearest neighbor (kNN) were used as spectral classifiers; standard normal variate (SNV) and norris derivative filtering (NDF) were applied for spectral preprocessing. The integrated algorithm combining separation degree priority combination (SDPC) with wavelength step-by-step phase-out (WSP) was utilized for the optimal wavelength selection.

**Results:**

The plasma spectral discriminant models for OBI and normal control were successfully established. Based on the optimal SNV-NDF preprocessed spectra, the SDPC-WSP-kNN and SDPC-WSP-PLS-DA methods determined the optimal number of wavelengths *N* to be 5 and 26, respectively. When evaluated on the independent external validation set, the SDPC-WSP-kNN model demonstrated better robustness, achieving sensitivity, specificity, and total recognition accuracy rates of 96.6%, 100%, and 98.7%, respectively. By introducing a grey judgment zone, both SEN and SPE reached 100%, with a detection recovery rate of 96.8%.

**Conclusion:**

These results indicated that Vis-NIR spectroscopy pattern recognition can accurately discriminate between OBI and normal controls’ plasma samples. This method is reagent-free, rapid, and simple, making it suitable for large-scale, low-cost rapid screening of OBI. In particular, the proposed few-wavelength model can provide an important reference for the development of small specialized blood analyzers for OBI detection.

## 1 Introduction

Hepatitis B virus (HBV) infection is a serious infectious disease and a global public health problem (Jeng et al., 2023). The main route of transmission of HBV is blood-borne (blood transfusions, blood products, etc.). Transfusion-transmitted HBV infection may lead to hepatitis, cirrhosis, hepatocellular carcinoma (HCC), and even death in patients (Justiz Vaillant et al., 2025). Preventing transfusion-transmitted infections (TTIs) is a priority in the management of conditions such as surgeries, and trauma (Candotti et al., 2019).

The progression of HBV infection is often jointly determined by the host’s innate and/or adaptive immune responses, which subsequently influence viral clearance, disease progression, and clinical outcomes. This process is accompanied by dynamic changes in serological markers, including HBV DNA, Hepatitis B surface antigen (HBsAg), and related antibodies (Hong et al., 2021; Kramvis et al., 2022). Occult hepatitis B virus infection (OBI) represents a specific form of HBV infection. Under the influence of multiple factors such as strong immune suppression, viral mutations, or low-level replication, OBI is characterized by undetectable HBsAg in blood despite the presence of HBV DNA, often with low and fluctuating viral loads (Raimondo et al., 2019; Huang et al., 2025). OBI is difficult to identify through routine HBsAg screening and is frequently missed. Importantly, although OBI is not currently incorporated into HBV treatment guidelines, it is considered a recognized key risk factor in transfusion medicine, as undetected HBV infection can lead to transfusion transmission (Raimondo et al., 2019). Studies have estimated that 8%–29% of transfusion-associated HBV infections may be attributable to OBI (Im et al., 2022). In contexts such as immunosuppressive therapy, chemotherapy, or organ transplantation, OBI may lead to viral reactivation and increase the risk of HCC (Peng et al., 2022; Schwarz et al., 2023; Hussein and Mahfodh, 2025). Thus, OBI detection is an essential component of HBV infection control (Ye et al., 2022). However, identifying OBI requires nucleic acid testing (NAT) for HBV DNA in addition to HBsAg screening (Ye et al., 2022; Saitta et al., 2022). In particular, in some low-income countries, NAT for OBI is not available in the blood collection/supply system, increasing the risk of OBI transfusion transmission (Cooke et al., 2024; Saravanan et al., 2024; Alshrari et al., 2025).

Visible-near-infrared (Vis-NIR) spectroscopy offers the advantages of rapid and straightforward analysis, holding significant potential for applications in the biomedical field (Wang et al., 2022). Blood samples, which are rich in clinical information, contain numerous components that exhibit characteristic absorption features in the Vis-NIR region. Vis-NIR spectroscopy has already been utilized for the quantitative analysis of several critical blood clinical indicators.

Spectral pattern recognition is based on the similarity within the same category of spectra and the differences between distinct categories, utilizing pattern recognition algorithms for classification. For classifying samples with similar compositions but low concentrations of characteristic components, spectral pattern recognition based on population characteristics is often simpler and more effective than quantitative analysis, which typically requires complex and expensive micro-quantitative analysis. Vis-NIR spectral pattern recognition represents a prominent application direction in the biomedical field (Wang et al., 2022)and has been successfully applied in clinical blood discrimination. These include the identification of human and animal blood (Fonseca et al., 2022), discrimination between β-thalassemia and normal controls (Yao et al., 2018), diagnosis of ovarian cancer from serum (Gajjar et al., 2013), classification of breast cancer and normal controls’ serum (Yuan et al., 2021), and identification of HBV-DNA excess in serum (Chen et al., 2023). These advances demonstrate the feasibility of using Vis-NIR spectroscopy for clinical blood classification.

Given that both OBI individuals and healthy individuals (normal controls) test negative for HBsAg, the accurate identification of OBI among HBsAg-negative blood samples is of critical importance. The fundamental distinction between plasma from OBI individuals and normal controls lies in the presence of HBV DNA in the blood of OBI individuals, which serves as a marker of viral replication after HBV infection.

The HBV DNA genome contains four open reading frames (ORFs)—S, C, P, and X—and nucleic acid testing for HBV often targets specific ORF fragments in the blood as detection markers (Saitta et al., 2022; Nguyen et al., 2020). In this study, the C and S gene fragments of HBV genome were amplified and tested by NAT. Any positive result for these gene fragments indicated OBI, whereas the normal controls were tested negative. According to the National Center for Biotechnology Information (NCBI) database, the primary amino acids encoded by the various HBV gene fragments include Proline (molecular formula C_5_H_9_NO_2_), Leucine (C_6_H_13_NO_2_), Serine (C_3_H_7_NO_3_), Alanine (C_3_H_7_NO_2_), Glycine (C_2_H_5_NO_2_), Arginine (C_6_H_14_N_4_O_2_), and others (Wu et al., 2022; Seeger and Mason, 2015). Compared with normal controls’ plasma, OBI samples exhibit altered levels of these amino acids. Furthermore, multi-omics studies have revealed significant differences between OBI individuals and normal controls in various metabolic pathways and enzyme activities, including protein metabolism, fatty acid metabolism, retinol metabolism, alcohol dehydrogenase (ADH), and UDP-glucuronosyltransferase (UGT) (Jiang et al., 2024).

The aforementioned amino acids, metabolites, and enzymes all contain hydrogen-containing groups (X-H), which exhibit characteristic absorption in the Vis-NIR spectral region. Therefore, this may lead to differences in the overall Vis-NIR spectral characteristics of OBI plasma compared with normal controls. Accordingly, establishing a discrimination model for OBI and normal control plasma samples using Vis-NIR spectral pattern recognition has a certain molecular mechanism basis. Subsequent experiments and modeling will indirectly validate this feasibility.

In recent years, near-infrared to mid-infrared (NIR-MIR) spectral pattern recognition has been applied to discriminate serum samples with elevated HBV DNA levels (Chen et al., 2023), providing preliminary evidence of an association between HBV DNA concentration and infrared spectral signals. Given that the presence of HBV DNA in the blood represents the key distinction between OBI and normal controls’ plasma, the aforementioned study indirectly supports the feasibility of applying Vis-NIR spectral pattern recognition for discriminating OBI from normal controls’ plasma samples. However, according to the literature search, there has not been reported about the spectroscopic discrimination of OBI and normal controls’ blood samples. Therefore, the development of a rapid Vis-NIR spectroscopy-based screening method for OBI in blood samples is of great significance for infectious disease prevention and control.

In this study, Vis-NIR spectroscopy combined with chemometrics was used to establish the discrimination models of the OBI and normal controls’ plasma samples. The research focused on the integrated optimization of classifier algorithms, spectral preprocessing methods, and multi-wavelength combination models for plasma spectral pattern recognition. Partial least squares-discriminant analysis (PLS-DA) (Wang et al., 2022; Chi et al., 2024) and k-nearest neighbor (kNN) (Mabwa et al., 2021; Ciloglu et al., 2020) were employed as classifier algorithms; standard normal variate (SNV) transformation (Barnes MSD and Lister, 1989; Dhanoa et al., 1994) and Norris derivative filtering (NDF) with multiple parameters (Yang et al., 2019; Norris, 2001) were applied for spectral preprocessing. A two-stage wavelength selection strategy was implemented: separation degree priority combination (SDPC) (Yuan et al., 2023; Pan et al., 2023)was used in the first stage, followed by wavelength step-by-step phase-out (WSP) (Yang et al., 2019; Tan et al., 2020) in the second stage. This study aims to develop a rapid, reagent-free, and accurate spectral pattern recognition method for screening occult hepatitis B virus infection.

## 2 Experiments and methods

### 2.1 Samples and reference methods

Plasma samples were collected from voluntary blood donors at the Nanning Blood Center in Guangxi, China. As part of routine screening, each plasma sample was initially tested twice for HBsAg by two independent operators using enzyme-linked immunosorbent assays (ELISA). To avoid detection variability, two different commercial ELISA kits (Bio-Rad, France; Wantai, Beijing, China; analytical sensitivity: 1 IU/mL) and detection systems (MICROLAB® F.A.M.E, Hamilton, Switzerland; URANUS AE 368, Aikon, China) were used. All samples that tested negative for HBsAg by ELISA were further confirmed using high-sensitivity electrochemiluminescence immunoassay (ECLIA; Roche Diagnostics, Germany; detection limit: 0.05 IU/mL) to rule out low-level HBsAg positivity. Only samples confirmed as HBsAg-negative by ECLIA were included in subsequent analyses. For HBV DNA testing, the cobas 6800 nucleic acid testing system (Roche Diagnostics, Germany; detection limit: 1.4 IU/mL) was used, targeting the C and S gene regions of the HBV genome. OBI was defined as the detection of HBV DNA in plasma in the absence of HBsAg (confirmed by high-sensitivity ECLIA). Healthy controls were defined as individuals negative for both HBsAg (confirmed by ECLIA) and HBV DNA.

Through the above rigorous testing procedures, a total of 204 OBI samples (166 males, 38 females) and 240 normal controls’ samples (180 males, 60 females) were collected. The discriminant criteria for OBI and normal controls are listed in Table 1.

**TABLE 1:** Sample categories based on HBsAg and HBV DNA detection.

Category/Test items	HBsAg	HBV DNA
Normal control	-	-
OBI	-	+

The test results are positive and negative, recorded as + and - respectively.

### 2.2 Experimental materials, instrumentation, and measurement methods

Spectra were acquired using a QE65Pro spectrometer (Ocean Optics, United States); Hamamatsu S7031-1006 thin back-illuminated CCD array detector was adopted; the spectral scanning range was 333–1118 nm (wavelength interval 0.79 nm, 1044 wavelengths); a 10 mm quartz cuvette was employed as the liquid transmission accessory. The integration time for spectral measurement was set to 8 ms, and the number of scans was set to 10. Each plasma sample was measured five times, and the average spectrum was used for subsequent spectral modeling and validation. All measurements were conducted under controlled ambient conditions (25 °C ± 1 °C, 46% ± 1% relative humidity).

### 2.3 Training-prediction-external validation procedure and model evaluation indexes

A rigorous training-prediction-external validation design was implemented. All plasma samples were first stratified into four subgroups according to category (OBI, normal control) and gender (male, female). Each subgroup was then randomly allocated to training, prediction, and independent external validation sets, which were subsequently pooled. The types and numbers of training, prediction, and external validation set samples are shown in Table 2. The training and prediction sets were used for modeling and parameter optimization. While in the external validation set, the independent samples that did not participate in modeling were used for model validation.

**TABLE 2:** Sample types and number assigned to the training, prediction, and external validation sets.

Type	Training	Prediction	External validation	Total
OBI				
Male	70	50	46	166
Female	15	10	13	38
Normal control				
Male	70	50	60	180
Female	15	10	35	60
Total	170	120	154	444

The performance evaluation indicators of the plasma discrimination model for OBI (positive) and the normal control (negative) are as follows: in the modeling process, the predicted category of each spectrum (positive, negative) was determined; by comparing the results with the clinically true categories, the positive, negative, and total recognition-accuracy rates ( ${\text{RAR}}_{T}^{+}$ ; ${\text{RAR}}_{T}^{‐}$ , ${\text{RAR}}_{T}$ ; ${\text{RAR}}_{P}^{+}$ ; ${\text{RAR}}_{P}^{‐}$ , ${\text{RAR}}_{P}$ ;%) on the training and prediction sets were calculated according to the following Formulas 1, 2:

$${\;{\text{RAR}}_{T}^{+}=\frac{{\tilde{N}}_{T}^{+}}{N_{T}^{+}},{\text{RAR}}_{T}^{‐}=\frac{{\tilde{N}}_{T}^{‐}}{N_{T}^{‐}},\text{RAR}}_{T}=\frac{{\tilde{N}}_{T}^{+}+{\tilde{N}}_{T}^{‐}}{N_{T}^{+}+N_{T}^{‐}},$$ (1)

$${\text{RAR}}_{P}^{+}=\frac{{\tilde{N}}_{P}^{+}}{N_{P}^{+}},{\text{RAR}}_{P}^{‐}=\frac{{\tilde{N}}_{P}^{‐}}{N_{P}^{‐}},{\;\text{RAR}}_{P}=\frac{{\tilde{N}}_{P}^{+}+{\tilde{N}}_{P}^{‐}}{N_{P}^{+}+N_{P}^{‐}}$$ (2)

Among them, the number of positive and negative spectra in the training and prediction sets by clinical diagnosis were ${\;N}_{T}^{+}$ , ${\;N}_{P}^{+}$ , $N_{T}^{‐}$ and $N_{P}^{‐}$ , respectively; the number of correctly identified spectra were ${\tilde{N}}_{T}^{+}$ , ${\tilde{N}}_{P}^{+}$ , ${\;\tilde{N}}_{T}^{‐}$ and ${\;\tilde{N}}_{P}^{‐}$ , respectively.

In the external validation process, the number of positive and negative spectra in the external validation set by clinical diagnosis were ${\;N}_{V}^{+}$ , $N_{V}^{‐}$ , respectively; the number of correctly identified spectra were ${\tilde{N}}_{V}^{+}$ , ${\;\tilde{N}}_{V}^{‐}$ , respectively. The corresponding number of incorrectly identified spectra (false negatives and false positives) were ${\;N}_{V}^{+}‐{\tilde{N}}_{V}^{+}$ and $N_{V}^{‐}‐{\tilde{N}}_{V}^{‐}$ , respectively. The sensitivity (SEN), specificity (SPE), false negative rate (FNR, i.e., missed diagnosis rate), and false positive rate (FPR, i.e., misdiagnosis rate) of the external validation set were calculated as the following Formula 3:

$$\text{SEN}=\frac{{\tilde{N}}_{V}^{+}}{N_{V}^{+}},\text{SPE}=\frac{{\tilde{N}}_{V}^{‐}}{N_{V}^{‐}},\text{FNR}=\frac{N_{V}^{+}‐{\tilde{N}}_{V}^{+}}{N_{V}^{+}},\text{FPR}=\frac{N_{V}^{‐}‐{\tilde{N}}_{V}^{‐}}{N_{V}^{‐}}$$ (3)

The total recognition-accuracy rate ( ${\text{RAR}}_{v}$ ) on the external validation sets was calculated, as the following Formula 4:

$${\text{RAR}}_{V}=\frac{{\tilde{N}}_{V}^{+}+{\tilde{N}}_{V}^{‐}}{N_{V}^{+}+N_{V}^{‐}}$$ (4)

### 2.4 Norris derivative filtering

Spectral preprocessing helps to correct noise interference such as spectral baseline-drift and tilt. Norris derivative filtering NDF is an effective multi-parameter spectral preprocessing algorithm (Yang et al., 2019; Norris, 2001; Pan et al., 2020), which includes moving average smoothing and difference derivation, and used three parameters: derivative order (*d*), number of smoothing points (*s*, odd number) and number of difference gaps (*g*). The specific calculation steps were as follows.

#### 2.4.1 Moving average smoothing

Smoothing window with *s* wavelengths was selected, s=1,3,⋯,S , $S\leq N_{0}$ , $N_{0}$ was the total number of wavelengths in the entire scanning region, *S* = 21 was set. In the smoothing window (center-symmetric), the absorbance of the central wavelength was replaced by the mean of all absorbances and the window was moved from left to right to complete the smoothing of the entire spectrum (except the leftmost and rightmost half-broadband wavelengths). The absorbance of the leftmost half-broadband wavelengths (leftmost $\frac{s‐1}{2}$ wavelengths) cannot be smoothed symmetrically. To ensure continuity, left-decreasing smoothing window $\left\{1,2,\cdots ,k+\frac{s‐1}{2}\right\}$ was used to smooth absorbance of kth wavelength $x_{k}$ , $k=1,2,\cdots ,\frac{s‐1}{2}$ . The calculation formula was as the following Formula 5 (Yang et al., 2019; Pan et al., 2020):

$$x_{k}=\frac{\sum_{i=1}^{k+\frac{s‐1}{2}}x_{i}}{k+\frac{s‐1}{2}},k=1,2,\cdots ,\frac{s‐1}{2}$$ (5)

Similarly, for the absorbance of the rightmost half-broadband wavelengths (rightmost $\frac{s‐1}{2}$ wavelengths), they can be smoothed using right-decreasing smoothing window.

#### 2.4.2 Difference derivative

Variable wavelength gap was used as the difference derivative gap (*g*), g=1,2,⋯,G , G=20 was set. For absorbance $x_{k}$ of *k*th wavelength, the 1st derivative ( ${Dx}_{k}$ ) of $x_{k}$ can be calculated by using central difference. The derivative values of all wavelengths were obtained by calculating from left to right (except the leftmost and rightmost half-broadband wavelengths). The absorbance of the leftmost half-broadband wavelengths (leftmost *g* wavelengths) cannot be differentiated symmetrically, and the 1st derivative was calculated by forward difference, as the following Formula 6 (Yang et al., 2019; Pan et al., 2020):

$$Dx_{k}=\frac{x_{k+g}‐x_{k}}{g},k=1,2,\cdots ,g$$ (6)

Similarly, for the absorbance of the rightmost half-broadband wavelengths (rightmost *g* wavelengths), the 1st derivative was calculated by backward difference.

Based on the above 1st derivative spectrum, the above difference derivation process was performed again to obtain the 2nd derivative spectrum, and so on. Considering that the absolute value of higher-order derivatives above the 3rd was small and the spectral information content was low, it was generally not recommended to use. The derivative order was set as d=0,1,2 . In particular, d=0 means no difference derivation, that is, only moving average smoothing was performed.

#### 2.4.3 NDF parameter optimization

For any parameter combination (*d*, *s*, *g*), there was a corresponding Norris derivative mode. For d=0,1,2 ; s=1,3,⋯,21 ; g=1,2,⋯,20 , there are a total of 11 + 2 × 11 × 20 = 451 algorithm modes. The three variable parameters with different functions made the Norris derivative spectra more diverse than the raw spectra, thus having a wider applicability. The NDF method was used to preprocess the plasma spectra. On this basis, the PLS-DA models corresponding to all NDF modes were established, and the optimal NDF parameters (*d*, *s*, *g*) were determined according to the recognition-accuracy rates on the prediction set ( ${\text{RAR}}_{P}$ ). In addition, the NDF preprocessed spectra were also applied to the kNN modeling.

### 2.5 Separation degree priority combination

For the given wavelength range (number of wavelengths: *n*), all wavelengths were represented in ascending order as: $\lambda_{1},{\;\lambda }_{2},\cdots ,{\;\lambda }_{n}$ . For any wavelength, the separation degree between the two types of spectra was calculated using the following Formula 7 (Yuan et al., 2023; Pan et al., 2023):

$$S\left(\lambda \right)=\left\{\begin{array}{c} A_{\mathrm{Min}}^{‐}\left(\lambda \right)-A_{\mathrm{Max}}^{+}\left(\lambda \right){,A}_{\text{Ave}}^{‐}\left(\lambda \right)\geq A_{\text{Ave}}^{+}\left(\lambda \right)\\ A_{\mathrm{Min}}^{+}\left(\lambda \right)-A_{\mathrm{Max}}^{‐}\left(\lambda \right){,A}_{\text{Ave}}^{‐}\left(\lambda \right)<A_{\text{Ave}}^{+}\left(\lambda \right) \end{array}\right.$$ (7)

The spectral separation degree quantified the discriminative capacity between two spectral populations. The wavelengths with higher separation degrees were expected to contribute more effectively to classification performance. Based on this principle, the Separation Degree Priority Combination (SDPC) method was proposed for wavelength selection, as described below: (Jeng et al., 2023): All wavelengths were sorted by separation degree values from largest to smallest, as follows: ${\tilde{\lambda }}_{1},{\tilde{\lambda }}_{2},\cdots ,{\tilde{\lambda }}_{n}$ ; (Justiz Vaillant et al., 2025); According to the priority of separation degree, the combination of the first i wavelengths from 1 to i was selected; there were a total of n combinations as follows: $\Omega_{i}=\left\{{\tilde{\lambda }}_{1},{\tilde{\lambda }}_{2},\cdots ,{\tilde{\lambda }}_{i}\right\},i=1,2,\cdots ,n$ ; (Candotti et al., 2019); Using the above n wavelength combinations, PLS-DA and kNN as the basis elemental methods, the SDPC-PLS-DA and SDPC-kNN integrated modeling algorithms were established, respectively. According to the predicted RAR_P_ of the model, the optimal i and the corresponding wavelength combination $\Omega_{i}$ was determined.

The determined optimal wavelength combination $\Omega_{i}$ is typically a combination of multi-waveband. The SDPC-PLS-DA and SDPC-kNN methods were employed in wavelength selection of the first stage.

### 2.6 Wavelength step-by-step phase-out

Wavelength step-by-step phase-out is a backward selection method (Yang et al., 2019; Tan et al., 2020). In an existing wavelength combination, by traversing all wavelengths, the interference wavelength with the best modeling effect is determined, based on the deleted remaining wavelength model. Phase-out based on the above rules until only one wavelength remains; the local optimal model obtained from each round was evaluated to comprehensively determine the global optimal model (according to the predicted RAR_P_ of the model, the optimal wavelength combination was determined). This method can effectively remove redundant wavelengths, and combined with the PLS-DA algorithm, WSP-PLS-DA has been established and successfully applied (Yang et al., 2019; Tan et al., 2020). In the current study, kNN was also used as a basic algorithm to establish the WSP-kNN model. The WSP-PLS-DA and WSP-kNN methods were used in the wavelength selection of the second stage.

The above-mentioned algorithms were constructed using MATLAB version 2020b software.

## 3 Results and discussion

### 3.1 Full spectral PLS-DA and kNN models with and without spectral preprocessing

**FIGURE 1:**
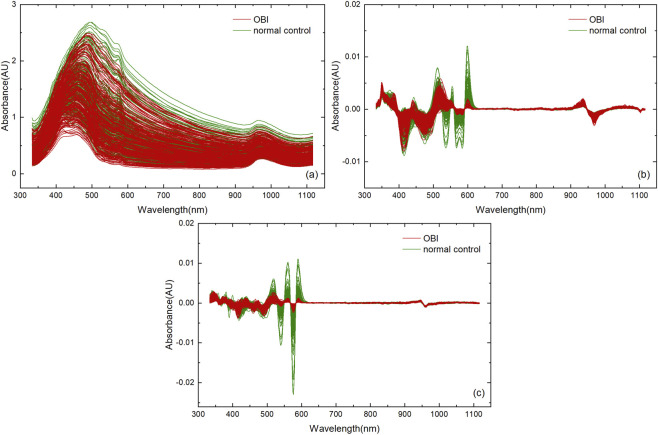
Vis-NIR spectra of all OBI and normal control plasma samples: **(a)** Raw spectra; **(b)** SNV-NDF combination preprocessing spectra (*d* = 2, s = 21, *g* = 2); **(c)** SNV-NDF combination preprocessing spectra (*d* = 2, *s* = 3, *g* = 3).

The Vis-NIR spectra of OBI and normal controls’ plasma samples in the full spectral range (333–1118 nm) are shown in Figure 1a. The green and red curves represent the spectra of the two types of samples, respectively. Significant baseline drift in the spectra was observed. Using raw spectra, PLS-DA and kNN discriminant models for OBI-normal control were established. Their training and prediction discrimination results are summarized in Tables 3, 4, respectively.

**TABLE 3:** Modeling effects of PLS-DA models for discriminating OBI-normal control with and without spectral preprocessing.

Method	*N*	Lv	${\text{RAR}}_{T}^{‐}$	${\text{RAR}}_{T}^{+}$	RAR_T_	${\text{RAR}}_{P}^{‐}$	${\text{RAR}}_{P}^{+}$	RAR_P_
Raw spectra	522	8	94.1%	100.0%	97.1%	85.0%	96.7%	90.8%
Preprocessed spectra	522	5	91.8%	100.0%	95.9%	93.3%	100.0%	96.7%

**TABLE 4:** Modeling effects of kNN models for discriminating OBI-normal control with and without spectral preprocessing.

Method	*N*	*k*	${\text{RAR}}_{P}^{‐}$	${\text{RAR}}_{P}^{+}$	RAR_P_
Raw spectra	522	3	73.3%	76.7%	75.0%
Preprocessed spectra	522	3	96.7%	100.0%	98.3%

The SNV was first used for spectral preprocessing; based on the SNV spectra, the NDF was used for further preprocessing, and the SNV-NDF combined spectra based on all the NDF modes were used to establish PLS-DA and kNN models. According to the optimal discriminative effect, the optimal Norris parameter combination was selected, and the optimal NDF parameter combinations for the PLS-DA and kNN models were *d* = 2, s = 21, *g* = 2, and *d* = 2, s = 3, *g* = 3, respectively. The two sets of preprocessed spectra are shown in Figures 1b,c, and the spectral baseline drifts were significantly improved. The corresponding training and prediction discrimination results are also summarized in Tables 3, 4, respectively. It can be seen that after spectral preprocessing, the RAR_P_ were improved substantially to 96.7% and 98.3%, respectively. However, the number of wavelengths *N* in the full spectrum was as high as 522, necessitating simplification of the wavelength model.

### 3.2 SDPC-PLS-DA and SDPC-kNN models

**FIGURE 2:**
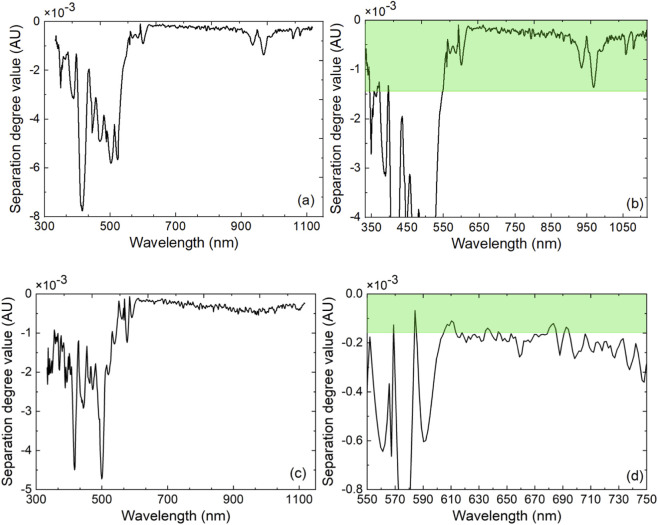
Separation degree spectrum of the two spectral populations after preprocessing in modeling: **(a)** 333–1118 nm (*d* = 2, *s* = 21, *g* = 2); **(b)** Selected waveband combination of the optimal SDPC-PLS-DA model (*d* = 2, *s* = 21, *g* = 2); **(c)** 333–1118 nm (*d* = 2, *s* = 3, *g* = 3); **(d)** Selected waveband combination of the optimal SDPC-kNN model (*d* = 2, *s* = 3, *g* = 3).

**FIGURE 3:**
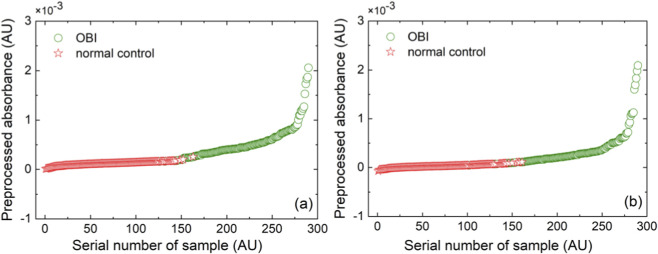
Preprocessed absorbance at a wavelength in order from smallest to largest for two types of modeling spectra: **(a)** 592 nm (*d* = 2, *s* = 21, *g* = 2); **(b)** 584 nm (*d* = 2, *s* = 3, *g* = 3).

Based on SNV-NDF spectra, the SDPC method was used to establish SDPC-PLS-DA and SDPC-kNN models to determine the wavelength combinations with the optimal discriminative effect. Using the separation degree method, the separation spectra (333–1118 nm) of the two types of modeling samples were calculated, as shown in Figures 2a,c. For the optimal SNV-NDF spectra for the PLS-DA and kNN models, the wavelengths with the highest separation degree, 592 nm and 584 nm, were taken as examples to observe the separation of the two types of spectra. The preprocessed absorbance values at 592 nm and 584 nm for all 290 modeling samples were sorted in ascending order and assigned new numbers. The preprocessed absorbance values for all samples are shown with new numbering in Figures 3a,b, where the red and green dots represent OBI and normal controls’ plasma samples. It can be clearly seen that the two types of samples are almost separated at wavelengths of 592 nm and 584 nm.

Using the SDPC-PLS-DA method, the optimal waveband combination (*N* = 398) selected was {333–344, 357, 366–373, 396, 550–1117} (nm), which can be seen in the green background part of Figure 2b, and the modeling discrimination effect is shown in Table 5. Using the SDPC-kNN method, the optimal waveband combination (*N* = 19) selected was {569, 584, 606–618, 635–638, 644, 680–685, 692–694} (nm), which can be seen in the green background part of Figure 2d, and the modeling discrimination effect is shown in Table 6. Compared with the full-spectrum model, the RAR_P_ of the optimal SDPC-PLS-DA and the SDPC-kNN models were both improved, while the numbers of wavelengths were greatly reduced.

**TABLE 5:** Prediction discriminant effect of optimal SDPC-PLS-DA models.

Waveband combination (nm)	*N*	Lv	${\text{RAR}}_{P}^{‐}$	${\text{RAR}}_{P}^{+}$	RAR_P_
333–344, 357, 366–373, 396, 550–1117	398	2	96.7%	100%	98.3%

**TABLE 6:** Prediction discriminant effect of optimal SDPC-kNN models.

Waveband combination (nm)	*N*	*k*	${\text{RAR}}_{P}^{‐}$	${\text{RAR}}_{P}^{+}$	RAR_P_
569, 584, 606–618, 635–638, 644, 680–685, 692–694	19	3	100%	100%	100%

The SDPC is a recently proposed novel spectral analysis method (Yuan et al., 2023; Pan et al., 2023). It calculates the separation spectra of two spectral populations based on the characteristics of the two types of sample spectra and sequentially combines the selected wavelengths in order of decreasing separation. The optimal wavelength combinations are determined according to the modeling performance, as shown by the green sections in Figures 2b,d, which correspond to the wavelength selections for the SDPC-PLS-DA and SDPC-kNN methods, respectively. Among these, 592 nm and 584 nm were the wavelengths with the greatest separation in the two aforementioned combinations. In Figure 3, 592 nm and 584 nm were used as examples to demonstrate the effective separation between the OBI and normal control spectral populations. Of course, information from a single wavelength is insufficient to support the classification in the OBI-normal control discriminant model. The optimal SDPC-kNN model achieved significantly better modeling performance (prediction recognition-accuracy RAR_P_ = 100%), with the corresponding wavelength combination of 584, 610, 612, 683, 607, 569, 682, 609, 692, 636, 680, 685, 606, 694, 638, 635, 644, 613, 618 (nm, *N* = 19, sorted by separation degree from high to low). According to literature reports, in the visible spectrum region, the absorption of hemoglobin (including oxyhemoglobin HbO_2_ and deoxyhemoglobin HHb) is mainly located in the 500–650 nm range (Prahl, 1999). In the current study, most of the selected wavelength combinations fall within the hemoglobin absorption bands, with the remainder corresponding to absorptions of other components, such as those related to lipid metabolism and bile acid metabolism.

### 3.3 SDPC-WSP-PLS-DA and SDPC-WSP-kNN models

The SDPC-based SDPC-kNN and SDPC-PLS-DA algorithms are forward optimization wavelength selection methods aimed at screening informative wavelengths; the WSP-based WSP-kNN and WSP-PLS-DA algorithms are backward optimization wavelength selection methods aimed at eliminating redundant wavelengths. SDPC and WSP were successively used in the first and second stages of wavelength selection. This wavelength selection strategy combined the advantages of forward optimization (selecting informative wavelengths) and backward optimization (eliminating redundant wavelengths). As a result, the integrated two-stage SDPC-WSP-kNN and SDPC-WSP-PLS-DA algorithms were used for modeling the plasma discrimination analysis of OBI-normal controls.

The WSP method was employed to further optimize the SDPC-PLS-DA and SDPC-kNN models. The number of wavelengths *N* of the optimal SDPC-WSP-PLS-DA and SDPC-WSP-kNN models significantly reduced to 26 and 5, respectively. Their modeling discrimination effects (RAR_P_) were substantially improved to 100%. As shown in Tables 7, 8.

**TABLE 7:** Prediction discriminant effect of the optimal SDPC-WSP-PLS-DA models.

Wavelengths (nm)	*N*	Lv	${\text{RAR}}_{P}^{‐}$	${\text{RAR}}_{P}^{+}$	RAR_P_
373, 603–604, 853–854, 955, 963–967,969, 973–979, 1048–1052, 1054,1073, 1100–1107	26	2	100%	100%	100%

**TABLE 8:** Prediction discriminant effect of the optimal SDPC-WSP- kNN models.

Wavelengths (nm)	*N*	*k*	${\text{RAR}}_{P}^{‐}$	${\text{RAR}}_{P}^{+}$	RAR_P_
569, 584, 613, 618, 644	5	1	100%	100%	100%

### 3.4 External validation of the models

Plasma samples from an external validation set that were not involved in the modeling process were used to verify the optimal discriminant models of SDPC-WSP-PLS-DA and SDPC-WSP-kNN for OBI and normal controls. The accuracy of the discriminant model was calculated based on the categories of the spectral discriminant and clinical diagnosis. For the optimal SDPC-WSP-PLS-DA and SDPC-WSP-kNN models, the sensitivity SEN, specificity SPE, false negative rate FNR, false positive rate FPR, and total recognition-accuracy rate ${\text{RAR}}_{v}$ on the external validation sets are summarized in Table 9. Among them, the ${\text{RAR}}_{v}$ of the two models reached 98.1% and 98.7%, respectively.

**TABLE 9:** Validation effect of the optimal SDPC-WSP-PLS-DA and SDPC-WSP-kNN models with SNV-NDF preprocessing.

Method	SEN	SPE	FNR	FPR	${\text{RAR}}_{v}$
SDPC-WSP-PLS-DA	96.6%	98.9%	3.4%	1.1%	98.1%
SDPC-WSP-kNN	96.6%	100%	3.4%	0%	98.7%

**FIGURE 4:**
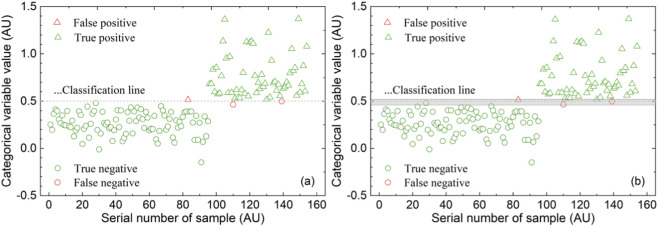
External validation results of the SDPC-WSP-PLS-DA model with SNV-NDF preprocessing: **(a)** Prediction value of class variable for OBI and normal control; **(b)** Schematic diagram of discrimination after introducing the gray area (*G*, 4.6 < *S* < 5.2).

**FIGURE 5:**
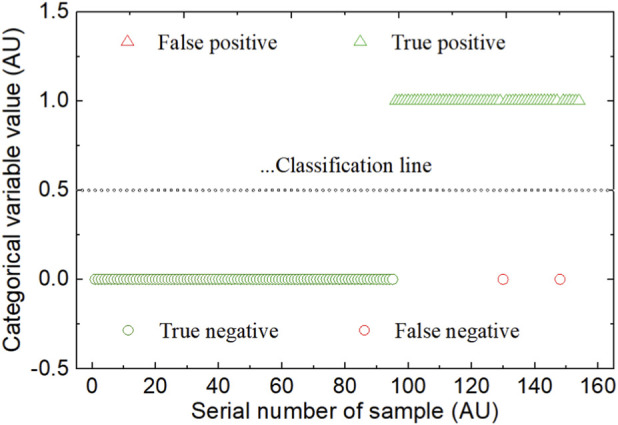
Prediction class variable labels in the SDPC-WSP-kNN model with SNV-NDF preprocessing.

For the SDPC-WSP-PLS-DA model with SNV-NDF preprocessing, the predicted class variable values (*S*, ≥0.5, <0.5) for OBI and normal controls’ spectra and the discrimination effect are shown in Figure 4a. For the SDPC-WSP-kNN model with SNV-NDF preprocessing, the predicted class variable labels (1,0) and the discrimination effect are shown in Figure 5.

Notably, for the PLS-DA method, the classification line for the categorical variables of the two sample classes was established, with a threshold of 0.5. Due to the diversity of samples and random interference, it is difficult to avoid misclassifying samples near the threshold. Therefore, during validation and application, a grey judgment zone *G* = {4.6 < *S* <5.2} was introduced, as shown in the gray area in Figure 4b. Considering that false negatives lead to missed diagnoses while false positives cause misdiagnoses, false negatives pose greater harm from the perspective of disease transmission risk. Thus, it is reasonable to set the negative portion of the gray zone wider. Based on this, the clear positive and negative judgment zones were defined as *D* ^+^ = {*S* ≥ 4.6}, *D* ^−^ = {*S* ≤5.2}, respectively. Under this configuration, out of 154 external validation samples, 5 samples fell into the gray zone *G* and will be further confirmed using other methods; the remaining 149 samples fall into the positive and negative zones *D* ^+^ and *D* ^−^, with both SEN and SPE reaching 100%, and FNR and FPR both at 0%. The method’s detection recovery rate reaches 96.8%.

The external validation results of both models demonstrated that plasma Vis-NIR spectral pattern recognition can be used to accurately discriminate between OBI and normal control.

In the current study, a real-time rapid OBI detection method based on plasma Vis-NIR spectral pattern recognition was developed. This method requires no reagents and represents an environmentally friendly green detection approach. The wavelength range for spectral measurement was 333–1118 nm, covering the entire visible and short-wave NIR spectral regions. Many components of clinical blood indicators have absorption characteristics within this spectral range (Nishadini Premasinghe et al., 2025). Moreover, photodetectors operating in this range can use high-efficiency and low-cost silicon (Si) detectors, making it possible to develop affordable related spectral systems, which facilitates the application of this technology for large-scale screening of HBV.

On the other hand, this study also proposes a high-precision detection model that requires only a few wavelength data points, providing a core solution for developing small OBI-specific spectral detection instruments. In recent years, with the rapid advancement of spectral detection equipment, the hardware cost in the visible to short-wave near-infrared spectral range has decreased significantly (even below 10,000 RMB), which is far lower than that of the main equipment currently used for OBI detection (nucleic acid testing). Moreover, this method does not require reagents, allows for rapid measurement, can instantly transfer spectral data to algorithm software, and displays results through embedded media, completing the entire process in just a few minutes. This is something that the vast majority of current clinical testing methods find difficult to achieve.

Due to the low cost of the hardware and the fact that no reagents are required, the corresponding testing costs will also decrease significantly. This makes the method suitable for rapid testing of large populations, which is difficult to achieve with nucleic acid methods. Of course, this method needs to be calibrated using standard clinical testing methods (hepatitis B surface antigen and nucleic acids). It cannot replace nucleic acid methods, but in terms of application, it is a novel and effective supplement, mainly in terms of rapid testing and suitability for large populations.

Regarding spectral pattern recognition, PLS-DA and kNN were used as the basic classifier algorithms. PLS combines the advantages of principal component analysis (PCA) and multiple linear regression (MLR). When combined with categorical variable discriminant analysis (DA), PLS-DA has demonstrated excellent modeling performance in many spectral discriminant analysis applications. kNN is a supervised learning method based on the Euclidean distance of spectral curves, offering the advantages of geometric interpretability and algorithmic simplicity. Combined with spectral preprocessing, kNN has also shown excellent modeling performance in many spectral discriminant analysis applications. In the current study, PLS-DA and kNN were used as the base classifier algorithms, and combined with wavelength selection, the plasma spectral discriminant models for OBI-normal control achieved 100% modeling accuracy, indicating that the choice of base algorithms was appropriate. On the other hand, once the wavelength combination model and the parameters of the base algorithms (*N*, *k*, Lv) are determined, the PLS-DA and kNN algorithms are very simple. These algorithms can be integrated into the embedded media of the spectrometer, allowing for rapid implementation of spectral measurements and discriminant model computation.

## 4 Conclusion

Occult hepatitis B virus infection individuals test negative for HBsAg, which requires additional HBV DNA testing for detection. Due to the relative complexity and cost of HBV DNA testing, it is typically excluded from general health examinations, leading to potential underdiagnosis of OBIs. In this study, Vis-NIR spectroscopy combined with novel chemometric methods was employed to establish a plasma discriminant analysis model for distinguishing OBI from normal controls in the HBsAg-negative population.

kNN and PLS-DA were employed as base classifiers; combination optimization of SNV-NDF was applied for spectral preprocessing; SDPC combined with WSP was used for two-stage wavelength selection. Consequently, the SDPC-WSP-KNN and SDPC-WSP-PLS-DA integrated optimization models were established and successfully applied for precise discrimination between OBI and normal controls’ plasma samples. Based on the optimal SNV-NDF spectra, the SDPC-WSP-KNN and SDPC-WSP-PLS-DA methods determined the optimal number of wavelengths *N* to be 5 and 26, respectively. Using independent external validation samples, the SDPC-WSP-kNN model demonstrated better robustness, with sensitivity, specificity, and total recognition-accuracy rate of 96.6%, 100%, and 98.7%, respectively. By introducing a grey judgment zone, both SEN and SPE reached 100%, with a detection recovery rate of 96.8%. In particular, the number of wavelengths used for modeling was smaller (*N* = 5), resulting in a simpler wavelength model.

The results indicate that plasma Vis-NIR spectral pattern recognition can be used for accurate discrimination between OBI and normal control. This method is rapid, reagent-free, and convenient, making it suitable for large-scale, low-cost rapid screening of OBI. The integrated modeling strategy proposed in this study, which considers both forward (SDPC) and backward (WSP) wavelength selection, may also apply to spectral analysis of other subjects. The proposed wavelength combination model can provide an important reference for the development of small dedicated blood analyzers for OBI.

## Data Availability

The raw data supporting the conclusions of this article will be made available by the authors, without undue reservation.
